# A convolutional recurrent neural network with attention for response prediction to repetitive transcranial magnetic stimulation in major depressive disorder

**DOI:** 10.1038/s41598-023-35545-2

**Published:** 2023-06-22

**Authors:** Mohsen Sadat Shahabi, Ahmad Shalbaf, Reza Rostami, Reza Kazemi

**Affiliations:** 1 Department of Biomedical Engineering and Medical Physics, School of Medicine, Shahid Beheshti University of Medical Sciences, Tehran, Iran; 2 Department of Psychology, University of Tehran, Tehran, Iran; 3 Department of Cognitive Psychology, Institute for Cognitive Science Studies, Tehran, Iran

**Keywords:** Network models, Biomedical engineering

## Abstract

Prediction of response to Repetitive Transcranial Magnetic Stimulation (rTMS) can build a very effective treatment platform that helps Major Depressive Disorder (MDD) patients to receive timely treatment. We proposed a deep learning model powered up by state-of-the-art methods to classify responders (R) and non-responders (NR) to rTMS treatment. Pre-treatment Electro-Encephalogram (EEG) signal of public TDBRAIN dataset and 46 proprietary MDD subjects were utilized to create time–frequency representations using Continuous Wavelet Transform (CWT) to be fed into the two powerful pre-trained Convolutional Neural Networks (CNN) named VGG16 and EfficientNetB0. Equipping these Transfer Learning (TL) models with Bidirectional Long Short-Term Memory (BLSTM) and attention mechanism for the extraction of most discriminative spatiotemporal features from input images, can lead to superior performance in the prediction of rTMS treatment outcome. Five brain regions named Frontal, Central, Parietal, Temporal, and occipital were assessed and the highest evaluated performance in 46 proprietary MDD subjects was acquired for the Frontal region using the TL-LSTM-Attention model based on EfficientNetB0 with accuracy, sensitivity, specificity, and Area Under the Curve (AUC) of 97.1%, 97.3%, 97.0%, and 0.96 respectively. Additionally, to test the generalizability of the proposed models, these TL-BLSTM-Attention models were evaluated on a public dataset called TDBRAIN and the highest accuracy of 82.3%, the sensitivity of 80.2%, the specificity of 81.9% and the AUC of 0.83 were obtained. Therefore, advanced deep learning methods using a time–frequency representation of EEG signals from the frontal brain region and the convolutional recurrent neural networks equipped with the attention mechanism can construct an accurate platform for the prediction of response to the rTMS treatment.

## Introduction

Major Depressive Disorder (MDD) disables a vast number of people worldwide and needs timely and effective treatment^1^. Initial pharmacological treatment shows partial effectiveness in depression, and finding a proper treatment follows trial and error among multiple antidepressants and treatments^2^. Some studies showed that this procedure gives remission to only one-third of MDD patients after 12 weeks of treatment initiation^3^. Repetitive Transcranial Magnetic Stimulation (rTMS) presented as a promising treatment for depressed patients to overcome this disorder. In this method successive strong magnetic pulses with usually 1–10 Hz frequency will be noninvasively applied to specific brain regions, especially the Dorsa-Lateral Prefrontal Cortex (DLPFC) for depression, in multiple sessions^4^. Many patients which not respond to one or two sessions of pharmacotherapy, can benefit from the rTMS method. However, some patients show poor responses to rTMS treatment. Therefore, the prediction of response to the rTMS treatment, can save multiple important weeks of the patient’s treatment. This can prevent the complication of disease by suicide attempts or medical comorbidities^5,6^. Therefore, the prediction of rTMS treatment outcomes is of paramount importance. Among different valuable techniques used for the prediction of response to rTMS, including clinical variables and neuroimaging modalities, Electro-Encephalogram (EEG) was used in multiple studies due to its availability and high clinical acceptance^7–9^.

Classification of pre-treatment EEG signals of responders and nonresponders to rTMS treatment can be facilitated by machine learning approaches that depend on extracted features. Erguzel et al.^10^ investigated the application of neural networks for the prediction of rTMS treatment outcome using the Cordance feature extracted from resting-state EEG of MDD patients. An accuracy of 89.09% was achieved for their shallow Neural Network which had 10 neurons in its one hidden layer. Hassanzadeh et al.^9^ used various features describing non-linear dynamics or power spectrum of different frequency bands of EEG signal for the K-Nearest Neighbor (KNN) classification method and correlation dimension feature obtained 93.5% accuracy for the prediction of response to rTMS treatment. Hassanzadeh et al.^11^ in a channel-wise study on the previous dataset showed that only the F8 EEG channel can produce 80% accuracy in the prediction of rTMS outcome. Shalbaf et al.^6^ used Empirical Mode Decomposition (EMD) to extract intrinsic Mode Functions (IMF) of EEG signals of 62 MDD patients and calculated the Permutation Entropy (PE) of these IMFs. Higher PE of second IMF in frontal channels observed and classification of responders and nonresponders to rTMS resulted in Area under the Curve (AUC) of 0.8. Arns et al.^7^ used a polygenic EEG approach for the prediction of response to rTMS treatment for MDD patients and discriminant analysis showed that this model can achieve an AUC of 0.735 based on functional connectivity measures calculated by fast Independent Component Analysis (FICA) of EEG data. Bailey et al.^12^ studied EEG connectivity measures of 42 MDD patients treated with rTMS and concluded that increased theta connectivity is a predictor of response to rTMS in the beginning or after the first week of treatment using a Support Vector Machine classifier (SVM). However, they studied the same hypotheses with a larger dataset (192 MDD subjects) and found that theta connectivity is not a treatment outcome predictor and their results were not replicated^8^. Corlier et al.^13^ investigated EEG functional connectivity and showed that the alpha spectral Correlation (αSC) feature based on Functional Connectivity in the alpha frequency band has predictive values in rTMS response prediction. Using αSC features as input to the ElasticNet machine learning model gained an AUC of 0.91.

As mentioned, machine learning methods are used for the classification of responders and nonresponders to rTMS based on features extracted from EEG signals by an expert person^14^. Recently, deep learning algorithms as a branch of machine learning introduced huge capabilities in the classification of images and time series^15,16^. Powerful deep learning models especially Convolutional Neural Networks (CNN) can automatically learn complex patterns laid in the data and the need for the special handcrafted features will be removed^17^. CNN models are specially constructed to extract optimal features from images and numerous studies use CNN models for EEG classification tasks^17^. However, Because of limitations in acquiring training data, especially in medical applications, the main obstacle against using deep learning models is the huge number of parameters that should be trained on the input data. Therefore, Transfer Learning (TL) models trained on a huge dataset beforehand, can be used to deal with limited training data by fine-tuning some parts of their parameters^18^. Jadhav et al.^19^ proposed a sleep stage classification model based on a pre-trained CNN model, named SqueezeNet, which can provide an overall accuracy of 84.74% for sleep stages using CWT images of EEG signals.

CNN models can not consider relationships between sequential inputs, then to obtain the most discriminative features from EEG signal, the temporal dependency between EEG samples have to be considered. Long-Term Short Memory (LSTM) cells are specially designed to extract temporal features from an input sequence and can be used with the CNN models to analyze a sequence of the input EEG samples in a spatiotemporal approach^20^. Saeedi et al.^21^ used hybrid deep learning models constructed using one and two-dimensional CNN (1DCNN-2DCNN) models and LSTM cells named 1DCNN-LSTM and 2DCNN-LSTM, respectively, to classify MDD and Healthy subjects. Using connectivity images obtained from EEG signals, the highest accuracy of their hybrid CNN-LSTM model reached 99.24%. Ay et al.^22^ constructed a hybrid CNN-LSTM model for depression detection using EEG signals of the right and left hemispheres of the brain and classified them with 99.12% accuracy. Additionally, some papers used two adjunct LSTM cells to create Bidirectional LSTM cells which can catch temporal dependencies in opposite directions of input sequences. Abdelhameed et al.^23^ used BLSTM cells on top of one-dimensional CNN models for seizure detection using the raw EEG signals and reached 98.89% accuracy in the classification of normal, interictal, and ictal cases. Khademi et al.^24^ utilized ResNet-50 and Inception-v3 architectures as transfer learning models in a hybrid CNN-LSTM approach for the classification of Motor Imaginary tasks based on EEG and gained 92% accuracy in the classification.

Handling long sequences of EEG signals can overload the memory of LSTM units which leads to overfitting of these models and the vanishing gradient has to be compensated in LSTM recurrent cells that work with a long input sequence. Multiple studies in EEG classification used the attention mechanism to overcome this problem in LSTM models by discarding redundant parts of data in long sequences of time-series data. Li et al.^25^ proposed a hierarchal attention-based temporal convolution network (HATCN) that uses intra-channel and inter-channel attention mechanisms for emotion recognition by extracting spectrograms from different EEG channels. This approach gained 71.6% accuracy for emotion recognition from the DEAP dataset. Singhal et al.^26^ developed a CNN-LSTM-Attention model to detect alcoholic subjects based on Fast Fourier Transform images of EEG signals to simultaneously capture spatiotemporal features of EEG signal and achieved 98.83% accuracy for the detection of alcoholism.

The aim of the present study was the development of a powerful deep learning model using state-of-the-art methods in neural networks to predict response to rTMS treatment using pre-treatment EEG signals. Time–frequency decompositions of EEG signals were provided using Continuous Wavelet Transform and were used by two powerful pre-trained CNN models named VGG16 and EfficientNetB0 to classify the responder and non-responder subjects. Indeed, a more powerful model was developed that concurrently deals with spatial and temporal features of EEG signals using Bidirectional LSTM recurrent units which were fed by a sequence of CWT images. Finally, TL-BLSTM models were equipped with an attention mechanism to extract the most discriminative patterns laid in long sequences of input EEG samples. All of these models were trained and evaluated on five brain regions named Frontal, Central, Temporal, Parietal, and Occipital to investigate the capabilities of each of these regions in developing a treatment outcome prediction platform.

## Methods

### Dataset

#### Proprietary data

46 MDD patients participated which were diagnosed as MDD by a psychiatrist based on DSM-IV criteria. Pre-treatment BDI-II (Beck Depression Inventory) questionnaire score was computed for each subject. Patients with suicidal risk and implants in the head and neck were excluded from this study. Each subject underwent 15 sessions (5 weeks) of rTMS treatment using Neuro MS (Neurosoft, Russia) with 10 Hz frequency which targeted the left DLPFC (Dorsa-lateral Prefrontal Cortex) for 37.5 min duration. It included 3000 magnetic pulses where each pulse is on for 4 s and off for 26 s. At the end of these sessions, the severity of depression of each subject was calculated by the BDI-II questionnaire, and response to treatment was defined as a more than 50% decrease in depression severity. Patients which showed response continued to the same protocol for 2 additional weeks and patients who does not show response continued for 2 weeks by a different protocol which was randomly selected to be either left 10 Hz, right 1 Hz, or bilateral rTMS. Based on this definition of response, 23 MDD patients responded to treatment and 23 MDD patients did not respond. Demographic data of responders and non-responders are shown in Table 1. The present study was approved by the Shahid Beheshti University of Medical Sciences ethics committee and then performed by following the rTMS Safety Guideline (2021), and all of the patients gave informed consent before treatment initiation.

**Table 1:** Demographic information of responders and non-responders from proprietary dataset and the TD-BRAIN dataset. ^a^AD (Antidepressant), MS (mood stabilizer), AP (antipsychotic). ^b^Medication type is not specified.

	Proprietary dataset			TD-BRAIN dataset		
	Non-responders	Responders	p-value	Non-responders	Responders	p-value
N	23	23		45	79	
Age	39 (± 14.6)	30.9 (± 12.3)	0.052	46.7 (± 13.9)	41.0 (± 12.2)	0.038
Gender (M/F)	8/15	8/15	0.9	26/19	38/41	0.30
Pre-treatment BDI-II	28.1 (± 9.4)	32.5 (± 9.3)	0.08	33.1 (± 11.4)	29.9 (± 8.9)	0.06
Post-treatment BDI-II	23.1 (8.4)	8.6 (± 5.9)	< 0.001	28.3 (10.8)	7.1 (± 5.5)	0.01
Illness duration (years)	7.9 (± 7.8)	6.5 (± 8.2)	0.278	–	–	–
Number of previous medications	2.7 (± 1.5)	2 (± 2.1)	0.108	–	–	–
Medications (AD/AD + MS / AD + MS + AP)^a^	5/8/1	6/6/0	X^2^ = 0.2, p = 0.65	15^b^	36^b^	–
Anxiety (Y/N)	14/9	19/4	p = 0.052	–	–	–

**Figure 1:**
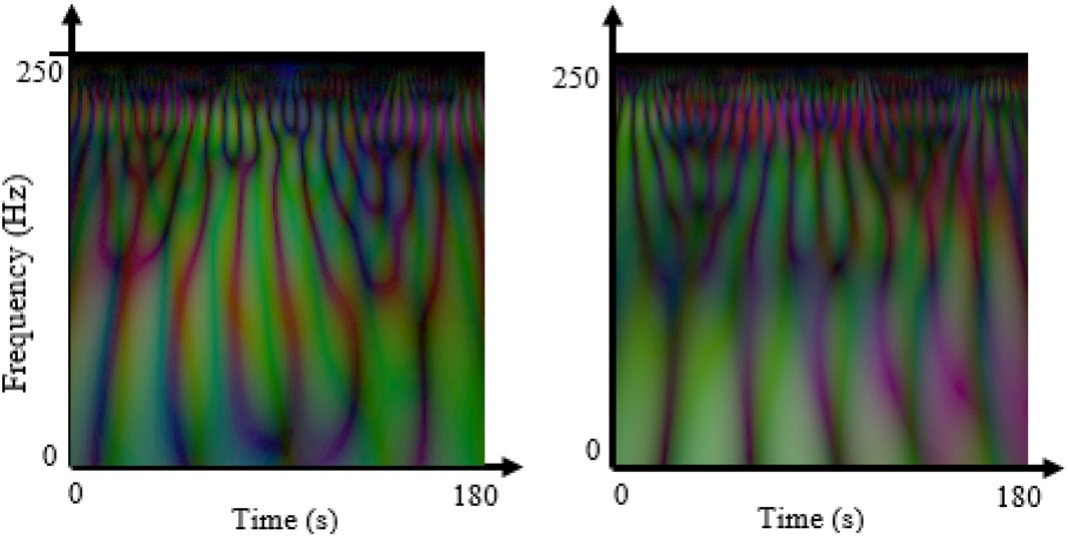
Example images of time–frequency representation of EEG signal of a responder subject (left) and a non-responder subject (right). The vertical (Y) axis represents the frequency in Hertz and the horizontal (X) axis shows the time in seconds.

EEG data were recorded in eyes closed situation for 5-min with linked-ear reference using 19-channel electrodes positioned according to the international 10–20 electrode positioning system which all electrodes designated as Fp1, Fp2, F7, F3, Fz, F4, F8, T3, C3, Cz, C4, T4, T5, P3, Pz, P4, T6, O1, and O2. EEG signal of five regions of the brain acquired using five sets of electrodes. The frontal region was measured by Fp1, F3, F7, Fz, Fp2, F4, and F8 electrodes and the parietal lobe was acquired by P3, Pz, and P4 electrodes. Measurement of the occipital region contains the signals from O1 and O2 electrodes, left and right temporal regions include T3, T4, T5, and T6 electrodes, and finally, C3, C4, and Cz electrodes are assigned to the central region of the brain. Pre-processing of the EEG signals has been done using EEGLAB software in MATLAB. To remove artifacts from EEG signals contaminated with 50 Hz power line noise, muscle movements, eye blinks and heart beats, a pre-processing pipeline was used. Firstly, a bandpass filter with low and high cut-off frequencies of 1 Hz and 42 Hz was applied to reject signals outside of this frequency band. Then MARA package from EEGLAB was utilized to remove noisy components of the EEG signal based on the Independent Component Analysis (ICA) technique. Finally, the remaining artifacts were removed by visual inspection. The sampling rate is 500 Hz and 3 min of EEG signal of each patient was utilized and the remaining data were discarded from the end of the EEG signal. The EEG signal was downsampled to 250 Hz for simplicity of creation of input images. Figure 1 shows a visual example of the time–frequency representation of the EEG signal of responders and non-responders.

#### TDBRAIN dataset

Two Decades-Brainclinics Research Archive for Insights in Neurophysiology (TDBRAIN)^27^ EEG database contains the resting-state EEG of 124 MDD patients (60 female/64 male with 18–73 years old) whose received the left DLPFC rTMS treatment. Pre-treatment EEG was acquired using 26 electrode channels which were positioned based on a 10–10 electrode international system at a 500 Hz sampling rate. Electrode collections representing Frontal, Central, Temporal, Parietal, and Occipital electrodes are designated as FP1, FP2, F3, F4, F7, F8, Fz, FC3, FC4, FCz, and C3, C4, Cz, CP3, CP4, CPz, and T5, T6, T7, T8, and P3, P4, Pz, and O1, O2, Oz, respectively. Pre-processing has been done using provided code by Van Dijk et al. which contains low and high frequency filtering, eye blink removal, and voltage jump and muscle artifact detection. Based on the definition of response to the treatment as the 50% decrease in the BDI-II score, 48 patients were non-responders and 72 patients were responders to the rTMS treatment.

### CNN models

The architecture of CNN models is based on hierarchical convolutions that take place in multiple layers and extract spatial features laid in the input images using the convolution of spatial filters with these images. In the light of back-propagation of gradients, the CNN model can obtain optimal patterns from input images, automatically. However, CNN models have a huge number of parameters to be trained and need a lot of training samples. This problem can be minimized using TL models which were trained on a huge dataset of ImageNet images and reduce the number of parameters that should be trained. By using this approach, the classification capabilities of validated pre-trained CNN architectures in image classification tasks can be used in EEG classification with a minimum burden^28,29^.

In the present study, VGG16 and EfficientNetB0 models which are designed to extract the best discriminative features from input images are utilized. VGG16 was created by a hierarchical architecture of convolutional layers which create multiple abstraction features from input images to provide the best classifiable representation of spatial features^30^. It uses 13 convolutional layers with 3 × 3 filters and 4 max-pooling layers which halve the size of feature maps and finally 3 fully connected layers on top of them. EfficientNet models were created based on an automatically optimized and scalable architecture to provide the best classification accuracy for input images. A specific EfficientNet architecture will be scaled in depth, width, and resolution by fixed scaling coefficients. EfficientNetB0 showed to achieve state-of-the-art results in image classification^31^. The parameters of these models should be tuned for being used in the current classification task of CWT images. Hyperparameters of each model, including batch size and learning rate, should be optimized to yield the best classification accuracy.

### LSTM cells

Long Short Term Memory cells proved their capabilities in the classification of time-series and especially EEG signals in various studies^21,32,33^. An LSTM unit can memorize long dependencies in input data by updating special weights and cell states based on new inputs and the last state of the cell. The current state of the LSTM cell is the central concept in controlling data flow in these units. Memory in LSTM cells depends on three different gates named input gate, forget gate, and output gate which contain trainable weights. A more advanced version of LSTM cells is bidirectional LSTM (BLSTM) which contains two LSTM cells that work in the opposite direction of each other. While one LSTM cell reads the input data from the beginning, another one reads the same data from the end, and the outputs of two LSTM cells will be concatenated. BLSTM cells extract the most complex patterns in input data and showed superior performance in multiple studies^23,34^.

### Attention mechanism

Attention mechanism introduced in natural language processing^35^ task and successfully used in EEG classification problems^34^. In long input sequences, when the gradient in the training phase vanishes by back-propagating to the first layers, some long dependencies with classification importance may be lost^34,36^. The attention mechanism can assure that the model did not lose important features from long sequences by assigning trainable weights to each input in a sequence. Attention weights will be low or high for irrelevant inputs and relevant ones, respectively. Bahdanau version^35^ of the attention mechanism used in this study.

### Proposed models

The architecture of the deep learning model in the present study is designed to fully capture spatiotemporal features from the time–frequency decomposition of the EEG signal obtained by CWT. For this aim, TL models which are powerful pre-trained CNNs get the WT images as input and create optimal abstract features for each CWT image and actually for each specific EEG segment. Then to fetch accurate temporal dependencies in the sequence of CWT images, which are equal to EEG signal segments, two BLSTM layers were added on top of CNN models. These recurrent layers will learn temporal patterns laid in spatial features of a sequence of input images. Finally, to signify those spatiotemporal features that help in the classification of responders and nonresponders, an attention mechanism layer was implemented in our model to maximize or minimize discriminative and non-discriminative features, respectively. To assess improvements gained by each step of development in model design, three models named TL models, TL-BLSTM models, and TL-BLSTM-Attention models were developed based on each pre-trained CNN model.

#### TL model

**Figure 2:**
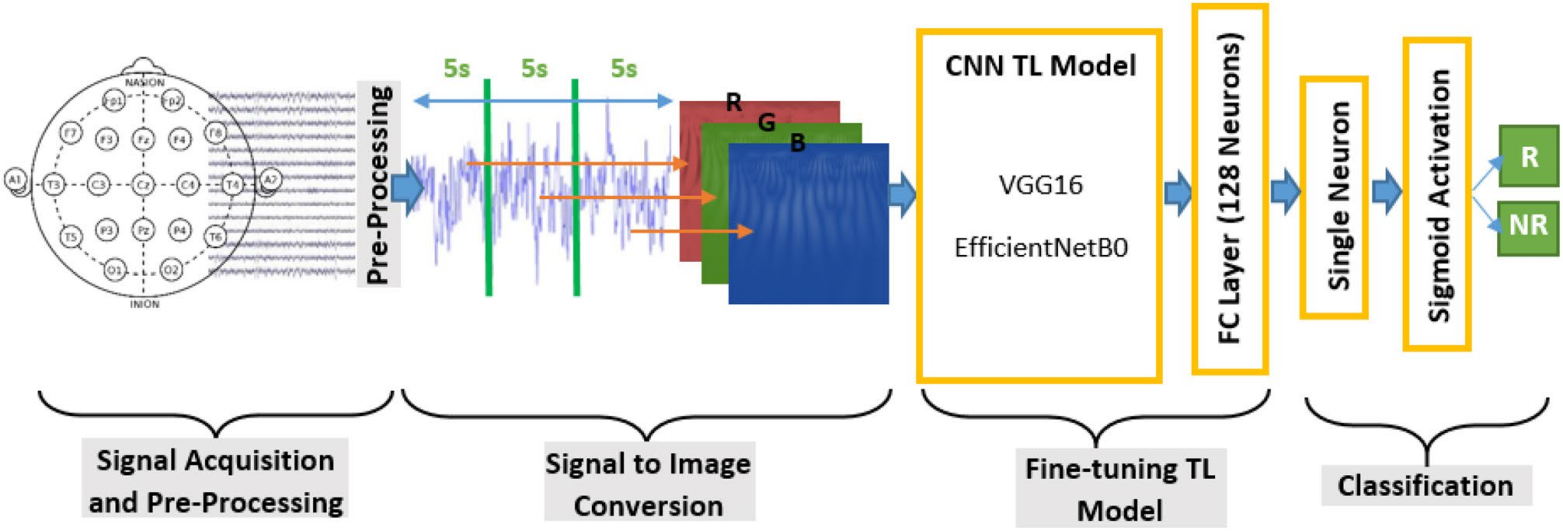
Block diagram of the TL model. After cleaning the noisy signal using pre-processing, three-dimensional RGB images were created from CWT of 5 s EEG segments. Pre-trained CNN models fine-tuned and classified CWT images as responders and non-responders.

The Block diagram of using TL models in the present study is illustrated in Fig. 2. To feed pre-processed EEG data to CNN models we can use a time–frequency representation of the input EEG signal which creates an informative two-dimensional image. Wavelet Transform as a powerful time–frequency transformation can deal with the non-stationary nature of EEG signal by extracting multiple scales in each time segment. WT was used to create input images for CNN models in multiple studies. Here, we used the Morlet mother function with a scale of 250 to create two-dimensional WT images. The EEG signal of each subject of each Dataset was divided into segments with 5 s duration (1250 samples of EEG signal) without overlapping. WT of each segment creates a two-dimensional image of size 250 × 1250 which will be resized to 224 × 224 to be fed into the CNN model. To investigate the importance and capability of each brain region in the prediction of response to rTMS treatment, WT images of different channels of a region were averaged to provide a single image in each time segment of EEG. For our Proprietary dataset, considering the 180-s duration of EEG data of each subject, there will be 36 images for each region. Then 36 WT images of five regions of brain data were appended together and shaped 5 × 36 image collection for each subject. The input layer of pre-trained CNN models used in this study accepts images with three channels, then we created an RGB image by concatenating three sequential images of a region. Each TL model should be trained and evaluated on all brain regions separately, therefore, (36/3) × 46 three-dimensional images (224 × 224 × 3) were created for train and evaluation of each CNN model. For the TDBRAIN dataset, there will be 24 images in each region, for 120 s of EEG signal duration. Therefore, (24/3) × 120 3D images (224 × 224 × 3) were created for train and evaluation of each CNN model on the TDBRAIN dataset. TL models used in this study are VGG16 and EfficientNetB0 which proved to be powerful models in image classification tasks. But, fine-tuning these models for adopting them with the present task as a binary classification of responders and nonresponders is necessary. To do this job, the main part of these models which are the convolutional and pooling layers should be kept and fully connected layers which reside on top of convolutional layers have to be discarded. In this work, for gaining the most capabilities of these pre-trained models, weights of convolutional layers obtained from pre-trained ImageNet weights and convolutional layers except the last convolutional layer freeze to be not trainable. Then a fully connected layer with 128 neurons equipped with the ReLu activation function is used to select the best distinguishing features as input of recurrent layers. For classification using TL models, a single neuron and sigmoid function were utilized.

#### TL-LSTM and TL-LSTM-attention

**Figure 3:**
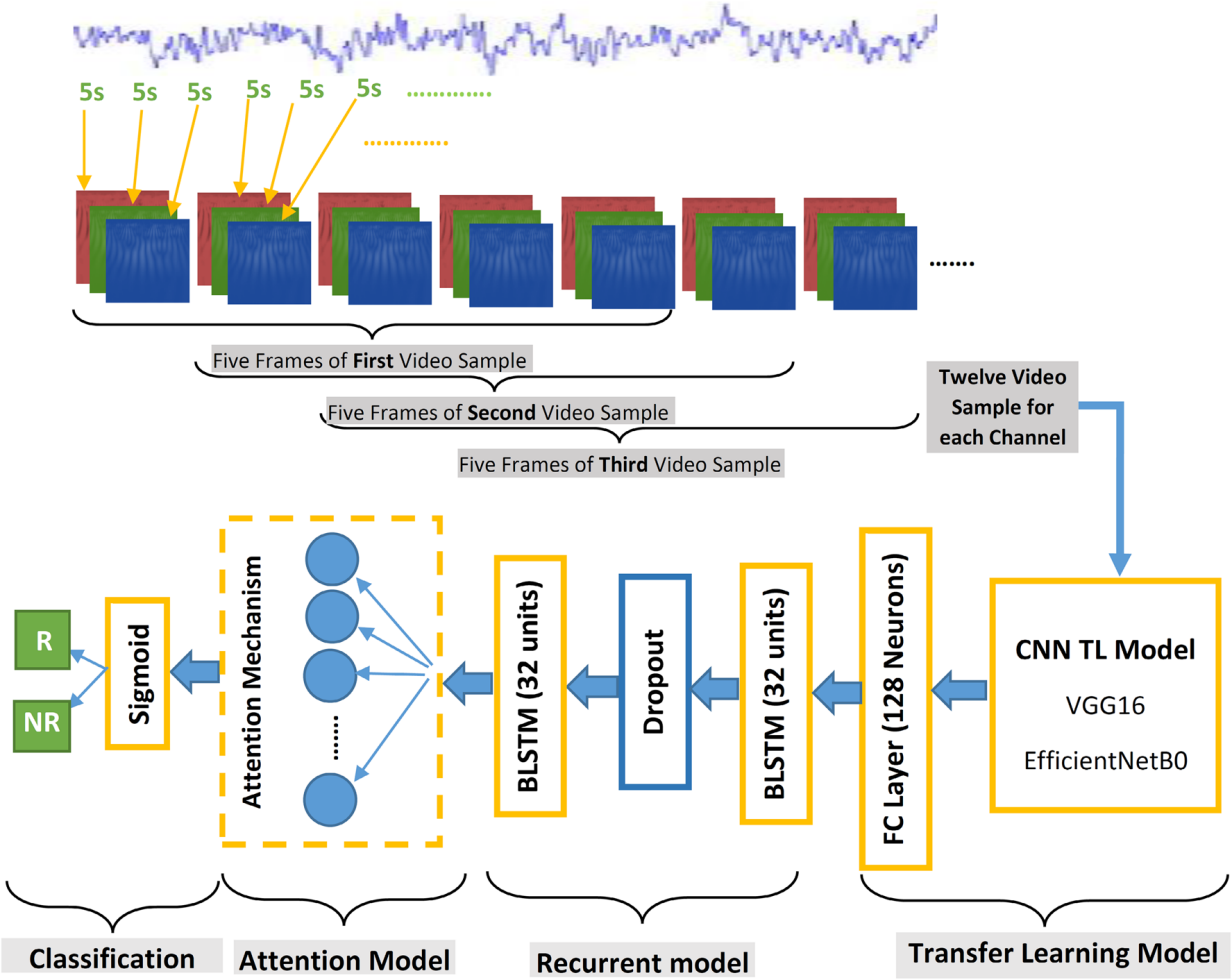
Block diagram of the TL-BLSTM and TL-BLSTM-attention model. Each five sequential RGB images create a video sample which will be fed into the TL models. A sequence of spatial features of each input image can be used as input to the recurrent model. Dashed block represents the attention block which can be added as an additional layer to the TL-BLSTM model to create the TL-BLSTM-attention model.

**Figure 4:**
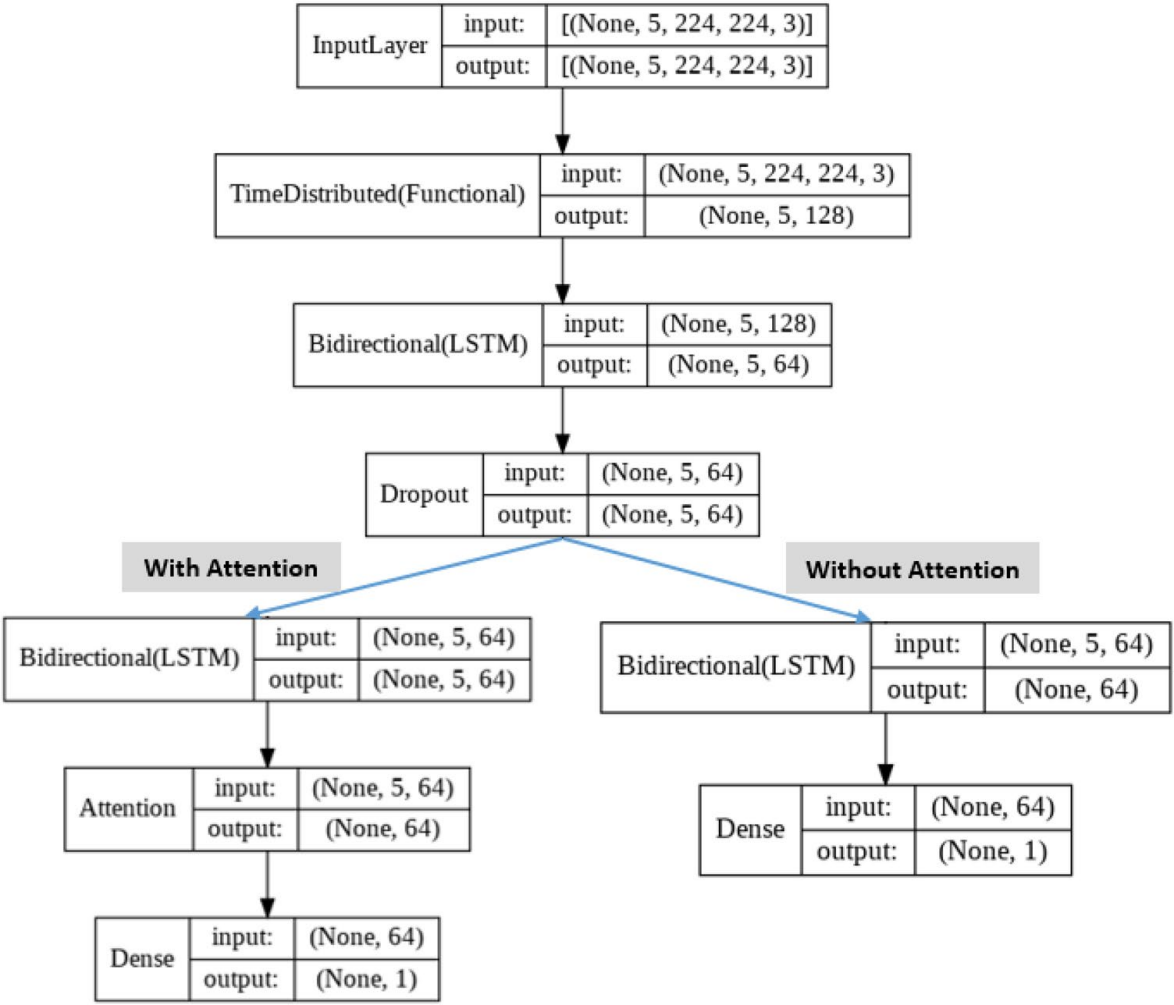
The architecture of the TL-BLSTM and TL-BLSTM-attention model was implemented in this study. Input video samples with the size of (5, 224, 224, 3) inserted to the model and spatial features with the size of (5, 128) extracted by TL models for each input sample. When attention is implemented, the second BLSTM layer should return a sequence of features with a size of (5, 64). Finally, the attention layer exports 64 most discriminative features to be classified.

To gain the power of recurrent networks such as LSTM, a sequence of input images must be provided. Therefore, we stacked each of five sequential 3D images of a brain region with a stride of 1 to create video samples including five frames (each frame is a 3D image). Finally, for our proprietary dataset, there will be 8 video samples for each of the five regions of the brain, which creates a total of 8 × 46 video samples to train and validate TL-BLSTM and TL-BLSTM-Attention models on each specific brain region. Similarly, there will be 8 video samples for each of the five regions of the brain, which creates a total of 4 × 120 video samples to train and validate TL-BLSTM-Attention models on each specific brain region of subjects from the TDBRAIN dataset. A schematic of the TL-BLSTM and TL-BLSTM-Attention models is presented in Fig. 3. Model construction continued by importing two BLSTM layers that each of which has 32 LSTM memory units. A dropout layer with a rate parameter of 0.3 was used between two BLSTM layers to decrease the overfitting problem. The first BLSM layer was set to return outputs of LSTM units in all time-steps with the shape of (5, 64) which were produced for five frames of a video sample and concatenated into two sets of outputs for 32 LSTM units. However, when we want to develop the TL-BLSTM model the second BLSTM can be set to not return sequences as output to produce (1, 64) feature vector which can be classified using a single neuron equipped by a sigmoid function (Fig. 3). To add the attention layer on top of these recurrent layers, and develop the TL-BLSTM-Attention model, the last BLSTM layer should return each intermediate output produced for frames of an input sample. Eventually, the attention layer tries to find relevant features among multiple frames of a video sample and returns a context vector that can be classified by a single neuron and sigmoid function. The dashed block in Fig. 3 which is called the Attention Module should be added to the TL-BLSTM model to construct the TL-BLSTM-Attention model. In this way, the last BLSTM layer will provide a sequence of rich features analyzed using TL-BLSTM as input to the attention layer which is illustrated as a separate branch in Fig. 4. Also, Fig. 4 shows the flow of input samples to the classification step.

The model development process has been done using python programming language and Keras package with Tensorflow backend. The attention layer is implemented by Subclassing the Layer class of Keras. Google Colaboratory platform which provides an NVIDIA Tesla K80 GPU with 12 GB RAM used to train and validate models. Adam optimizer and binary cross-entropy loss function were utilized to train all kinds of models in 50 epochs. Batch size and learning rate which are the main hyper-parameters in our study are optimized for each model and each input dataset to gain the highest value of accuracy in the classification of responders and nonresponders. The same hyperparameters were used for TL-BLSTM and TL-BLSTM-Attention models. Table 2 shows the optimized hyper-parameters of the models.

**Table 2:** Hyperparameters tuned for each base TL model.

Model name	Batch size	Learning rate
VGG16	20	1e−5
EfficientNetB0	16	2e−5

### Performance evaluation

From 46 subjects who participated in this study, 2760 images and 1840 video samples were created. To evaluate the performance and generalizability of TL, TL-BLSTM, and TL-BLSTM-Attention models on every single region of the brain, each model was separately trained and evaluated on WT images and video samples of that single region of the brain. The number of images and sample videos of each brain region are 552 and 368, respectively. We randomly split 20 percent of our dataset as the test data and the remaining 80 percent data were used for the training. In the training phase, we used k-fold cross validation (k value set to be 10) to train and evaluate the model and tune the hyper-parameters of our model using just the training data. It means that all training subjects were divided into k parts and each model’s training phase was performed on the k − 1 part of the data and validated on one remaining part to tune the hyper-parameters of our model. This process is repeated k times to use on all parts of training data for model development and evaluation. Using this method, we can be sure that the model development process used all training data. Finally, the trained and tuned models were tested using the test data and the results were calculated as the performance of the proposed models on the previously unseen test data. Therefore, TL, TL-BLSTM, and TL-BLSTM-Attention models were evaluated using this process and the test data was not seen in the model training phase.

## Results

Three deep learning models named TL, TL-LSTM, and TL-LSTM-Attention were developed to use WT images obtained from pre-treatment EEG signals of MDD patients for the prediction of response to rTMS treatment. After pre-processing the EEG signal of each subject, WT was used to produce time–frequency decomposition images from different segments of the EEG signal. Sequences of five 3D wavelet images, named video samples, were created for investigating the most discriminative spatiotemporal features from EEG signals which correspond to responders and nonresponders. TL-LSTM-Attention models proposed in this study are based on powerful pre-trained CNN models, named VGG16 and EfficientNetB0 to provide the best classification results. To consider the impact of the EEG signal of each region on the brain, all TL and TL-BLSTM or TL-BLSTM-attention models were trained independently on images and video samples obtained from each brain’s region, respectively.

**Figure 5:**
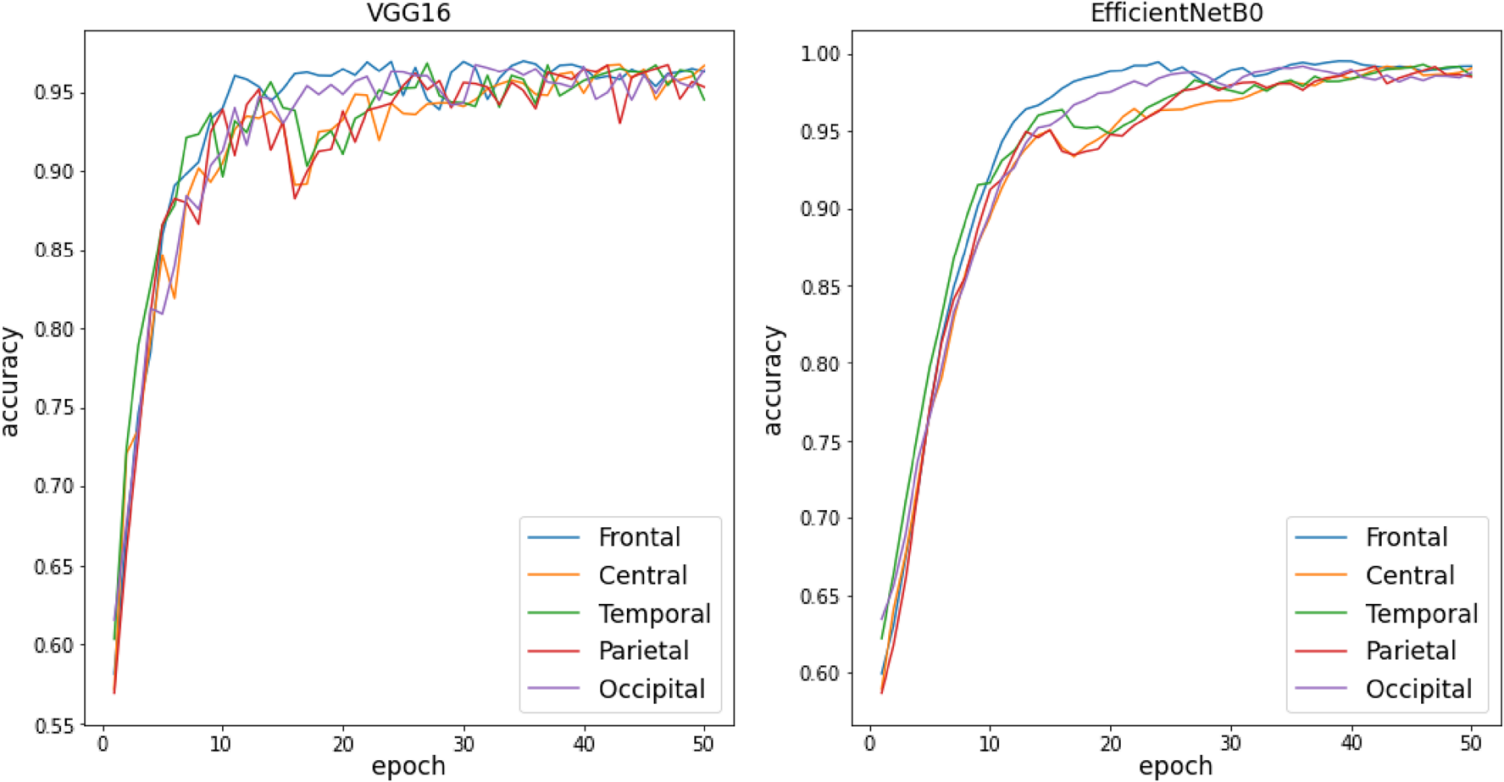
Validation accuracy and loss curves for TL models used for classification of responders and non-responders. Five regions of the brain are named Frontal, Central, Temporal, Parietal, and Occipital distinguished by colors specified in the legend of the figure.

**Figure 6:**
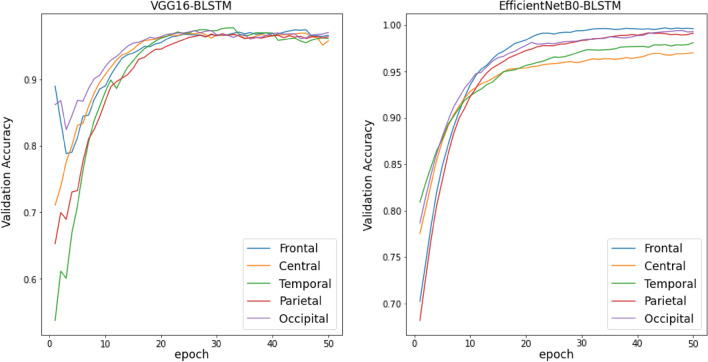
Validation accuracy and loss curves for TL-BLSTM models used for classification of responders and non-responders.

**Figure 7:**
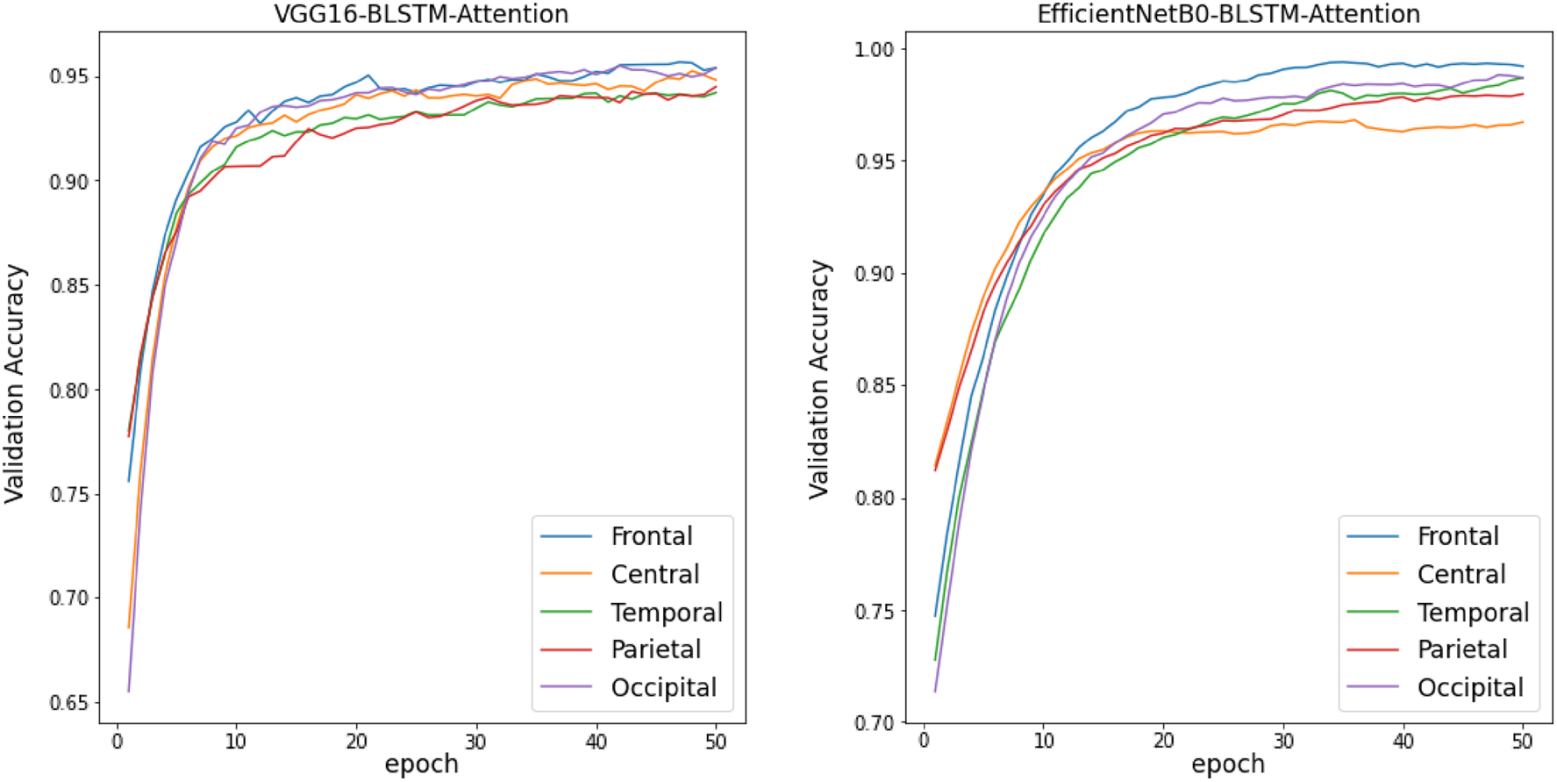
Validation accuracy and loss curves for TL-BLSTM-attention models used for classification of responders and non-responders to rTMS treatment.

Training Curves of all TL, TL-BLSTM, and TL-BLSTM-attention models are presented in Figs. 5, 6, and 7. The accuracy and loss of different models implemented for each specific brain area are compared with each other in 50 epochs of training. Each curve represents an average plot of 10 folds of the cross-validation method. According to Fig. 5, the TL models which were trained using input data from the Frontal brain region provided superior performance and more confident results compared to the models trained on other brain areas, because of minor fluctuations in training curves. Also, TL-BLSTM (Fig. 6) and TL-BLSTM-attention models (Fig. 7) showed the process of learning spatiotemporal features by increasing the accuracy of the validation data in each epoch.

We explored each of the two pre-trained CNN models as base TL models and developed TL-BLSTM and TL-BLSTM-attention extensions for each of these base models. The performance of all models using data from each brain region is provided in Tables 3 and 4 which show that EfficientNetB0-BLSTM-attention trained by frontal EEG data has the best performance among all models and brain regions investigated in this study. The average and standard deviation of Accuracy (ACC), Sensitivity (SEN), and Specificity (SPE) of all models were calculated for 10 folds of the cross-validation method. Table 3 shows the results for models based on the VGG16 TL model. The highest performance measures were obtained for models trained by data from the frontal brain region. VGG16, VGG16-BLSTM, and VGG16-BLSTM-attention models have the highest accuracy of 89.2%, 95.2%, and 96.8% for the frontal brain region, respectively. Also, the second-highest scores were recorded for the Occipital area Models based on EfficientNetB0 which was trained by data from the Frontal EEG signals showed improvement in results with the highest accuracies of 91.2% for EfficientNetB0, 96.2% for EfficientNetB0-BLSTM, and 97.1% for EfficientNetB0-BLSTM-Attention models, respectively. Performance of TL, TL-BLSTM, and TL-BLSTM-Attention models based on VGG16 for different brain regions on unseen test data. ACC, SEN, and SPE stand for accuracy, sensitivity, and specificity, respectively. The mean and standard deviation of each performance measure is presented. According to Table 4, the EfficientNetB0-BLSTM-Attention model achieved the best sensitivity, specificity, and AUC of 97.3%, 97.0%, and 0.96 for EEG of the frontal region. In most experiments, sensitivity was higher than the accuracy and specificity of that model. Also, the lowest performance in nearly all models was calculated for temporal and central brain regions.

**Table 3:** Performance of TL, TL-BLSTM, and TL-BLSTM-attention models based on EfficientNetB0 for different brain regions on unseen test data. The mean and standard deviation of each performance measure is presented.

		Frontal	Central	Temporal	Parietal	Occipital
VGG16	ACC (%)	89.2 ± 4.6	86.5 ± 3.3	86.9 ± 4.1	87.1 ± 3.8	88.9 ± 4.5
	SEN (%)	90.2 ± 3.8	86.4 ± 3.0	88.3 ± 2.5	89.4 ± 2.8	90.3 ± 3.5
	SPE (%)	89.5 ± 3.4	86.0 ± 3.5	85.2 ± 3.1	85.5 ± 3.5	86.4 ± 3.7
	AUC	0.88 ± 0.03	0.85 ± 0.04	0.84 ± 0.05	0.86 ± 0.06	0.86 ± 0.05
VGG16-BLSTM	ACC (%)	95.2 ± 2.9	93.6 ± 1.9	92.2 ± 2.5	93.9 ± 2.4	94.4 ± 2.9
	SEN (%)	95.5 ± 1.5	95.4 ± 2.7	93.4 ± 2.9	94.5 ± 2.6	95.3 ± 3.0
	SPE (%)	94.9 ± 3.1	93.2 ± 2.8	91.8 ± 2.9	92.5 ± 2.1	93.9 ± 2.3
	AUC	0.95 ± 0.03	0.91 ± 0.03	0.93 ± 0.05	0.92 ± 0.03	0.94 ± 0.04
VGG16-BLSTM-Attention	ACC (%)	**96.8 ± 2.4**	95.2 ± 0.9	94.5 ± 1.0	94.7 ± 2.0	96.1 ± 1.7
	SEN (%)	**97.7 ± 1.2**	95.4 ± 1.0	95.1 ± 1.5	95.5 ± 1.6	96.6 ± 0.9
	SPE (%)	**96.4 ± 1.4**	94.8 ± 1.6	94.1 ± 1.5	94.5 ± 1.6	95.3 ± 0.8
	AUC	**0.96 ± 0.03**	0.94 ± 0.02	0.93 ± 0.04	0.94 ± 0.04	0.95 ± 0.03

Significant values are in bold.

**Table 4:** Performance of TL, TL-BLSTM, and TL-BLSTM-attention models based on EfficientNetB0 for different brain regions on unseen test data. The mean and standard deviation of each performance measure is presented.

		Frontal	Central	Temporal	Parietal	Occipital
EfficientNetB0	ACC (%)	91.2 ± 3.7	87.6 ± 3.5	88.8 ± 4.0	89.3 ± 3.2	91.2 ± 4.1
	SEN (%)	91.2 ± 3.5	89.1 ± 2.8	90.9 ± 3.4	90.7 ± 3.7	91.9 ± 3.1
	SPE (%)	89.2 ± 3.4	86.5 ± 2.9	86.0 ± 3.0	88.4 ± 4.0	89.7 ± 3.2
	AUC	0.90 ± 0.05	0.88 ± 0.05	0.88 ± 0.04	0.88 ± 0.04	0.90 ± 0.02
EfficientNetB0-BLSTM	ACC (%)	96.2 ± 2.3	94.2 ± 1.8	94.5 ± 2.1	94.7 ± 2.6	95.8 ± 2.8
	SEN (%)	96.7 ± 2.2	94.5 ± 2.1	94.8 ± 2.0	95.5 ± 2.7	96.3 ± 3.4
	SPE (%)	95.8 ± 3.1	93.5 ± 2.8	93.2 ± 2.9	94.3 ± 2.1	95.0 ± 2.3
	AUC	0.95 ± 0.03	0.93 ± 0.04	0.94 ± 0.04	0.95 ± 0.03	0.96 ± 0.03
EfficientNetB0-BLSTM-Attention	ACC (%)	**97.1 ± 1.1**	95.6 ± 0.9	95.3 ± 1.0	95.7 ± 1.4	96.3 ± 0.7
	SEN (%)	**97.3 ± 0.8**	96.0 ± 1.2	95.9 ± 1.2	96.5 ± 1.8	97.1 ± 1.0
	SPE (%)	**97.0 ± 1.2**	95.5 ± 2.2	94.9 ± 1.5	95.0 ± 1.6	96.8 ± 0.9
	AUC	**0.96 ± 0.01**	0.95 ± 0.02	0.95 ± 0.03	0.96 ± 0.03	0.96 ± 0.02

Significant values are in bold.

**Figure 8:**
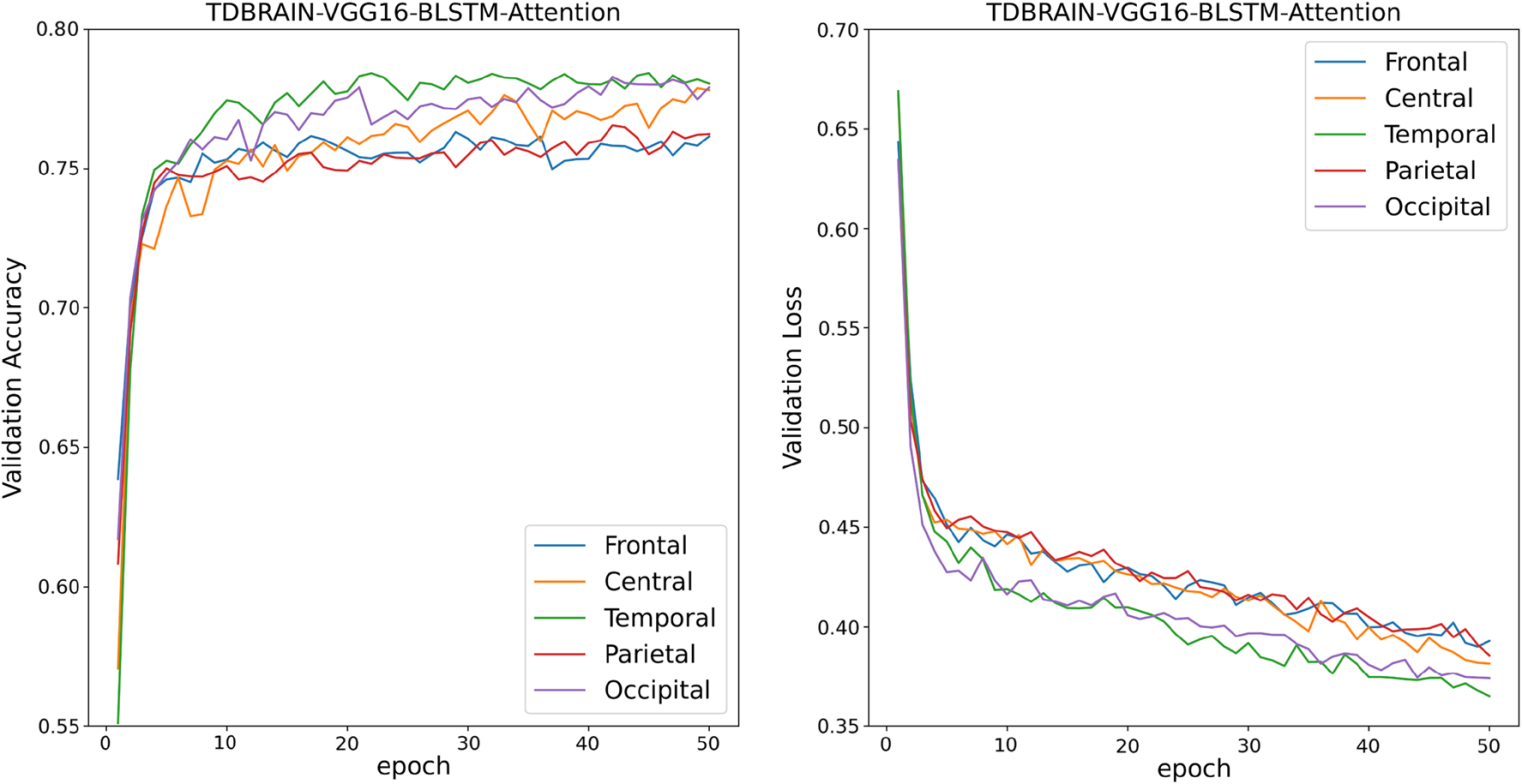
Validation accuracy and loss curves for VGG16-BLSTM-attention models used for classification of responders and non-responders to rTMS treatment of TDBRAIN dataset.

**Figure 9:**
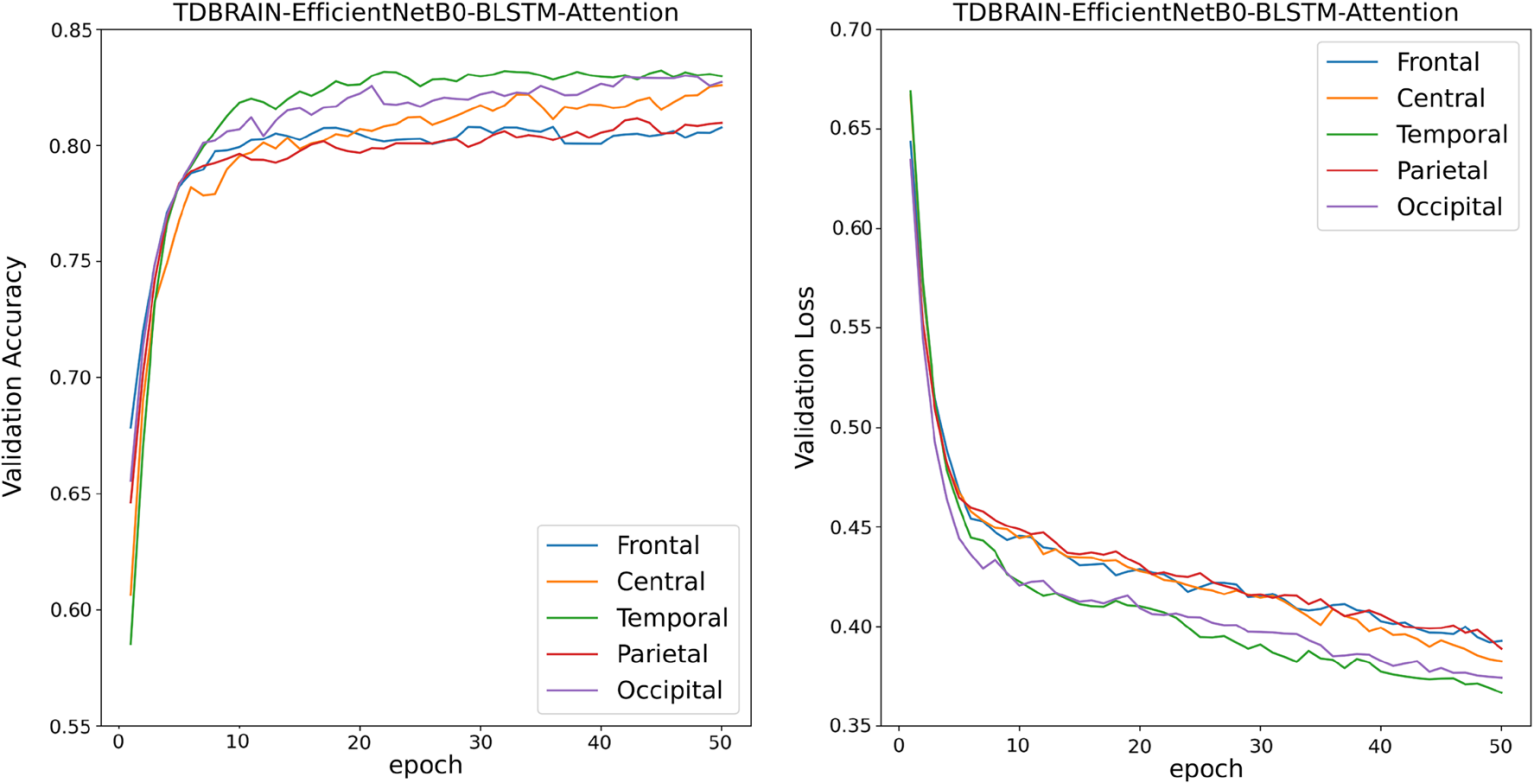
Validation accuracy and loss curves for EfficientNetB0-BLSTM-attention models used for classification of responders and non-responders to rTMS treatment of TDBRAIN dataset.

Figures 8 and 9 shows the learning curves of TL-BLSTM-attention models for five brain regions using the TDBRAIN dataset. All models converged in 50 epochs but show lower performance compared to the TL-BLSTM-Attention models developed based on our proprietary data. In these models which are trained on the TDBRAIN dataset, the Temporal region provides the highest performance measures. The efficientNetB0-BLSTM-Attention model showed the highest performance and gained an accuracy of 82.3%, a sensitivity of 80.3%, a specificity of 81.9%, and an AUC of 0.83. Table 5 shows the detailed performance measures of the VGG16-BLSTM-Attention and the EfficientNetB0-BLSTM-Attention models for five brain regions.

**Table 5:** Performance of TL-BLSTM-attention models based on EfficientNetB0 and VGG16 for different brain regions on unseen test data of TD-BRAIN dataset. The mean and standard deviation of each performance measure is presented.

		Frontal	Central	Temporal	Parietal	Occipital
VGG16-BLSTM-Attention	ACC (%)	81.0 ± 4.2	79.2 ± 5.6	81.7 ± 4.1	79.9 ± 4.6	78.4 ± 3.8
	SEN (%)	77.8 ± 3.5	77.4 ± 4.1	74.1 ± 3.0	79.5 ± 3.7	76.6 ± 4.4
	SPE (%)	81.9 ± 3.4	80.5 ± 3.7	82.5 ± 3.6	80.4 ± 3.1	78.5 ± 3.5
	AUC	0.82 ± 0.05	0.80 ± 0.04	0.83 ± 0.05	0.79 ± 0.02	0.79 ± 0.03
EfficientNetB0-BLSTM-Attention	ACC (%)	80.8 ± 3.7	80.7 ± 2.6	**82.3 ± 3.3**	79.7 ± 4.8	80.2 ± 3.2
	SEN (%)	79.5 ± 1.9	78.1 ± 3.2	**80.2 ± 3.4**	78.3 ± 3.8	79.2 ± 2.9
	SPE (%)	81.0 ± 3.0	81.2 ± 3.7	**81.9 ± 2.8**	79.0 ± 1.6	79.9 ± 3.1
	AUC	0.82 ± 0.01	0.81 ± 0.03	**0.83 ± 0.03**	0.79 ± 0.05	0.79 ± 0.03

Significant values are in bold.

## Discussion

In the present study, we aimed to predict response to rTMS treatment using pre-treatment EEG signals and developed multiple deep learning models based on state-of-the-art methods in this field. A time–frequency representation of EEG segments yielded by CWT as an input to powerful transfer learning models to decompose the embedded local patterns of EEG signal. To fully extract temporal dependencies in EEG signals, chronological sequences of these images are obtained to be fed into the hybrid TL-BLSTM model. Finally, an attention layer is implemented on top of this hybrid model to prevent losing long-term relations in spatiotemporal features of sequential EEG segments. Two pre-trained CNN models named VGG16 and EfficientNetB0 were used as base TL models to learn the most valuable spatial features from each CWT image and prepare them for the subsequent bidirectional recurrent layers which are BLSTM layers. Attention layers will eventually select the best context for classification. Therefore, we implemented three types of models named TL, TL-BLSTM, and TL-BLSTM-Attention based on the selection of each pre-trained CNN as a TL model. Additionally, each model proposed in this study is independently developed using EEG signals of each brain region including the Frontal, Central, Temporal, Parietal, and Occipital areas for the prediction of rTMS treatment outcome. Two datasets including a proprietary dataset and a public dataset called TDBRAIN utilized in this study. Superior results were obtained by TL-BLSTM-Attention models based on two different TL models. According to Tables 3 and 4, the best classification performance of all models was achieved by using data from the frontal brain region of the proprietary dataset. The efficientNetB0-BLSTM-Attention model had the best performance with an accuracy of 97.1 ± 1.1%, a sensitivity of 97.3 ± 0.8%, a specificity of 97.0 ± 1.2%, and an AUC of 0.96 ± 0.03. Stability and generalizability are important aspects in the selection of models with applicability to new test data. Less variance in model performance on new unseen test data may help to more confidently use these models.

CWT images obtained from EEG, firstly represent a simplified and indeed, powerful map of time and frequency bindings in small segments of signal and secondly prepare 2D inputs for extracting the most distinctive spatial patterns from input data. Xu et al.^37^ successfully applied WT on the EEG signal of the BCI competition II dataset and reached 90% classification accuracy using pre-trained CNN models. Morlet mother function which is used in this paper is a Gabor-like function that produces smooth images because of the non-orthogonality and Gaussian behavior of this base function. Numerous works used CWT based on the Morlet mother function for EEG classification with interesting results^38–40^.

The main contribution of our work was integrating two BLSTM layers and attention mechanism with pre-trained CNN models for a joint investigation of spatiotemporal features of EEG signals. Using two layers of BLSTM upon base TL models, classification performance improved by about 5–7 percent. This increment in performance can be better understood when presented by classification error. From this point of view, using BLSTM models halves the classification error of base TL models. This interesting capability of the BLSTM cell is because of the special gates of the LSTM cell and recovering long dependencies in the backward direction of the input sequence using the second LSTM cell. Multiple studies showed the huge potential of BLSTM layers in EEG classification^41,42^. Finally, the attention layer was imported to select relevant and irrelevant spatiotemporal features extracted by TL-BLSTM models for the classification of responders and nonresponders to rTMS treatment. This layer acts as a simple weighting layer by multiplying each input feature vector by a trainable weight. Then, it can select the most important features in a long sequence to prevent the loss of useful features and just with a minimum increase in model parameters. Attention mechanism has been used for the classification of EEG and multi-modal data of depression and caused the robustness and generalizability of the model^36^.

The development of models based on the EEG signal of each brain’s region, not only can show the robustness of the deep learning architecture proposed in this study but also reveals the classification capabilities of each underlying brain area. As shown in Tables 3 and 4, the EEG signal of the frontal region yielded the highest classification accuracy among different regions of the brain. The acceptable performance of these models demonstrates the power of the deep learning models used in the present study. Many papers reported that most discriminative features of EEG signals for the prediction of rTMS treatment outcome belong to frontal brain regions. Hasanzadeh et al.^11^ showed that the combination of power and complexity features of the F8 channel is the most powerful predictor of responders and nonresponders to rTMS using single-channel EEG. Shalbaf et al.^6^ reported that the permutation entropy of the second IMF extracted from frontal channels of pre-treatment EEG provides the highest classification performance in treatment outcome prediction. Corlier et al.^13^ Investigated the functional connectivity changes in frontal channels and concluded that connectivity variations in the frontal region of the brain are associated with rTMS treatment outcome.

Table 6 summarized some of the reports that used machine learning approaches for the prediction of response to rTMS treatment. Comparison of the number of subjects, extracted features, classification algorithm, and model performance presented. Our work using state-of-the-art methods in deep learning models, including TL models, BLSTM cells, and attention mechanism provided a high classification performance for rTMS treatment outcome using pre-treatment EEG signal.

**Table 6:** Comparison with some of the papers that used machine learning approaches for rTMS treatment outcome prediction against methods and results. R and NR stand for responders and non-responders.

study	Number of subjects	Methods	Performance
Erguzel^10^—2015	147 MDD (90 R/57 NR)	Cordance feature + ANN	ACC = 89.09%
Bailey^12^—2018	39 MDD (10 R/29 NR)	Working memory-related fronto-midline theta power and Theta connectivity + SVM	ACC = 91%
Erguzel^43^—2016	147 MDD (90 R/57 NR)	Cordance + ANN, SVM, Decision Tree	AUC = 0.92
Shalbaf^6^—2018	51 MDD (30 R/21 NR)	Permutation entropy + ANOVA	AUC = 0.8
Hasanzadeh^9^—2019	46 MDD (23 R/23 NR)	Correlation dimension, fractal dimension, power and Bispectrum + KNN	ACC = 93.5%
Corlier^13^—2019	109 MDD (45% R/55% NR)	Functional connectivity + ElasticNet	AUC = 0.91
Bailey^8^—2021	193 MDD (128 R/65 NR)	Theta connectivity + ANOVA	No statistically difference
Our work	46 MDD (23 R/23 NR)	CWT images of frontal electrodes + TL-BLSTM-attention	ACC = 97.1%

The main limitation of the present study was the low number of participants in the proprietary dataset. Therefore, a large public dataset was utilized to validate the model’s performance and show that the results are not because of the overfitting of the proposed model on the proprietary dataset. Multiple methods are implemented to overcome the overfitting problem in the present study. The segmentation of the EEG signal for the creation of multiple input samples for each subject and each brain region, Transfer Learning models based on Convolutional Neural Networks which were pre-trained using the ImageNet dataset, and dropout layer with 0.5 ratio were used to handle the overfitting problem. However, there is still a large gap between the model’s performance on the proprietary dataset and the TDBRAIN dataset. Another limitation of this work was that the model’s decisions were not explainable. Therefore, a user can not recognize why and based on which features the model reached a specific decision. This limitation will be our future direction of research to move from black box models which are known as non-interpretable models to the white box models which are known as fully interpretable models. The clinical utility and explainability of the proposed models have to be explored to help in the decision making process, especially when there is a difference between the doctor’s decision and the model’s prediction. To this aim, we plan to implement an interpretable model combined with an ensemble method to create an XAI (Explainable Artificial Intelligence) framework in our future work.

## Conclusions

Using advanced deep learning methods in the present study including BLSTM layers and attention mechanism alongside powerful pre-trained CNN models, named VGG16 and EfficientNetB0 resulted in a new deep learning framework that reached an accuracy of 97.1%, a sensitivity of 97.6%, specificity of 96.8% and AUC of 0.96 in the classification of responders and nonresponders to rTMS treatment using pre-treatment EEG signal. Also, the evaluation of the proposed models using the TDBRAIN dataset gained the highest accuracy of 82.2% and the AUC of 0.83. Time–frequency decomposition images produced by CWT of EEG time segments can be helpful in the automatic feature extraction by TL models. A region-based study of these advanced convolutional recurrent models showed that high accuracy of treatment outcome prediction is achievable just using EEG electrodes of the frontal brain region. Therefore, an accurate combination of powerful methods in deep learning models can be used for providing superior results in rTMS response prediction.

## Data Availability

The proprietary dataset used and analysed during the current study is available from the corresponding author on reasonable request.
